# A2BR Adenosine Receptor Modulates Sweet Taste in Circumvallate Taste Buds

**DOI:** 10.1371/journal.pone.0030032

**Published:** 2012-01-10

**Authors:** Shinji Kataoka, Arian Baquero, Dan Yang, Nicole Shultz, Aurelie Vandenbeuch, Katya Ravid, Sue C. Kinnamon, Thomas E. Finger

**Affiliations:** 1 Rocky Mountain Taste & Smell Center, University of Colorado School of Medicine, Aurora, Colorado, United States of America; 2 Department of Cell & Developmental Biology, University of Colorado School of Medicine, Aurora, Colorado, United States of America; 3 Depatment of Otolaryngology, University of Colorado School of Medicine, Aurora, Colorado, United States of America; 4 Departments of Medicine and Biochemistry and Whitaker Cardiovascular Institute, Boston University School of Medicine, Boston, Massachusetts, United States of America; The Research Center of Neurobiology-Neurophysiology of Marseille, France

## Abstract

In response to taste stimulation, taste buds release ATP, which activates ionotropic ATP receptors (P2X2/P2X3) on taste nerves as well as metabotropic (P2Y) purinergic receptors on taste bud cells. The action of the extracellular ATP is terminated by ectonucleotidases, ultimately generating adenosine, which itself can activate one or more G-protein coupled adenosine receptors: A1, A2A, A2B, and A3. Here we investigated the expression of adenosine receptors in mouse taste buds at both the nucleotide and protein expression levels. Of the adenosine receptors, only A2B receptor (A2BR) is expressed specifically in taste epithelia. Further, A2BR is expressed abundantly only in a subset of taste bud cells of posterior (circumvallate, foliate), but not anterior (fungiform, palate) taste fields in mice. Analysis of double-labeled tissue indicates that A2BR occurs on Type II taste bud cells that also express Gα14, which is present only in sweet-sensitive taste cells of the foliate and circumvallate papillae. Glossopharyngeal nerve recordings from A2BR knockout mice show significantly reduced responses to both sucrose and synthetic sweeteners, but normal responses to tastants representing other qualities. Thus, our study identified a novel regulator of sweet taste, the A2BR, which functions to potentiate sweet responses in posterior lingual taste fields.

## Introduction

In the peripheral gustatory system, ATP plays a crucial role in the transmission of information from taste buds to the gustatory nerve fibers [1], [2], [3], [4]. ATP is released from taste receptor cells and activates ionotropic ATP receptors (P2X2/P2X3) on taste nerves [5], [6] as well as metabotropic (P2Y) receptors on taste cells [7], [8], [9]. The importance of purinergic transmission is evidenced by the loss of essentially all gustatory neural responses in P2X2/P2X3 double knockout (KO) mice [1].

Mature taste cells can be classified into three distinct types based on morphologic, molecular, and functional features [10]–[20]. Type I “glial-like” cells, originally termed “dark cells” based on ultrastructural criteria, are similar in some ways to astrocytes; they envelop the other cell types without obvious functional contacts and express the ectoATPase, ectonucleoside triphosphate diphosphohydrolase 2 (NTPDase2) and the glial glutamate/aspartate transporter (GLAST) [19], [21] which serve as molecular markers for this cell type [22]. Type II “receptor” cells, originally termed “light cells”, express the G protein-coupled receptors for umami (T1R1/T1R3), sweet (T1R2/T1R3) or bitter (T2Rs) [23], [24] transduction. These taste receptors are expressed in largely non-overlapping subsets of Type II (also called “receptor”) taste cells, but all couple to the same downstream signaling effectors including phospholipase C-β2 (PLCβ2), inositol 1, 4, 5-trisphosphate receptor type 3 (IP3R3) and transient receptor potential channel M5 (TrpM5) [22]. Thus these signaling proteins serve as well-characterized markers of Type II taste cells in all taste fields. The close correspondence between Type II cell ultrastructure and expression of these signaling components has been established for both PLCβ2 [25] and gustducin [26], [27]. The Gα subunits vary according to receptor type and location on the tongue. While bitter and umami receptors generally couple to GαGustducin (Gαgust) in both anterior and posterior tongue, taste cells that express sweet receptors co-express Gαgust in anterior tongue and Gα14 in posterior tongue [28], [29]. Type III (also termed “presynaptic”, [22]) cells were originally classified as “intermediate cells” because they have ultrastructural features intermediate between Type I and Type II cells. Type III cells are responsible for sour taste transduction [30], and express several definitive markers including NCAM [31], PKD2L1 [20], and carbonic anhydrase isoenzyme 4 (Car4) [32]. Type III cells accumulate and release several transmitters, including 5-HT, GABA, and noradrenalin [9], . Whereas Type III cells form classical synapses onto the intragemmal nerve fibers, Type II cells do not [13], [35]. Type II cells do closely associate with nerve fibers, but lack conventional synapses [13], [25], [26], and instead release ATP via gated hemichannels [2], [3] to activate purinergic P2X2 and P2X3 receptors on afferent nerve fibers [1].

The action of extracellular ATP is terminated by the characteristic ectonucleotidase within taste buds, NTPDase2 expressed by Type I taste cells [19]. NTPDase2 degrades ATP to form ADP which can then act on local purinergic P2Y receptors [9], or be broken down further by NTPDase2 and by ecto-5′ nucleotidases and other phosphatases [19], [36]–[41]. The end product of the phosphatase activity will yield adenosine, which itself can activate one or more G-protein coupled adenosine receptors: A1R, A2AR, A2BR, and A3R. Here we investigated the expression of adenosine receptors in mouse taste buds. Of the adenosine receptors, A2BR is expressed in taste buds of the posterior tongue, and there, only in the subset of taste cells that contain the sweet taste receptors. Functional recordings from glossopharyngeal nerves of A2BR KO mice show significantly reduced sweet taste responses compared to wildtype (WT) littermates. These data suggest that A2BR functions to potentiate transmission of sweet taste information in posterior lingual taste fields.

## Materials and Methods

### Animals

Most experiments relied on C57BL/6J mice except where otherwise indicated. A2BR-KO mice (on a C57BL/6 background) were generated in Boston University [42], [43]. In this line, Exon 1 of the A2BR gene was replaced by β-galactosidase (β-gal), thus animals homozygous for the transgene had genetic deletion of A2BR protein but carried the β-gal marker in cells expressing from the A2BR locus. Hemizygous animals, herein referred to as A2BR-lacZ retained expression of the native A2BR protein but also expressed the marker in the relevant cell populations. Some of the tongue tissue obtained from these animals, as described below was shipped to Denver from Boston Univ. for immunohistochemical analysis. Other animals were bred and utilized entirely from stocks maintained at the University of Colorado. The University of Colorado Health Science Center Institutional Animal Care and Use Committee approved the use of these animals in all studies (Protocol B-07610(02)1E). In all experiments we used animals of both sexes in the age range of 2–9 months for control, A2BR-lacZ and A2BR KO mice.

### RT-PCR

Adult C57BL/6J mice (Jackson Labs) of both sexes 2–4.5 months old were euthanized by CO_2_ asphyxiation followed by cervical dislocation. The tongue was removed and a protease mixture consisting of 2.5 mg/ml dispase, 1 mg/ml collagenase, type A, (both from Roche Products, Indianapolis, IN) and 1 mg/ml trypsin inhibitor (Sigma, St. Louis, MO) in Tyrode's buffer was injected under the epithelium. Tyrode's buffer consisted of 139 mM NaCl, 5 mM KCl, 2 mM CaCl_2_, 1 mM MgCl_2_, 10 mM Hepes, 10 mM glucose, 10 mM Na pyruvate, and 5 mM NaHCO_3_; pH 7.2, 318–323 mOsm. Tongues were incubated in Ca/Mg-free Tyrode's solution for 40 min. For Ca/Mg-free Tyrode's buffer, CaCl_2_ and MgCl_2_ were replaced with 2 mM EGTA and 2 mM BAPTA.

Circumvallate (CV) epithelium was peeled from underlying tissue and placed directly into RNAlater (Applied Biosystems/Ambion, Austin, TX) for storage. To avoid possible contamination of taste cells, epithelium was also peeled from the ventral surface of the tongue for a non-taste tissue control. Mouse brain total RNA was purchased from Clontech (Mountain View, CA). Total RNA was purified using the Qiagen Micro RNeasy Kit (Qiagen, Valencia, CA) according to manufacturers instructions, and incubated with DNase I to remove any contaminating genomic DNA. First strand cDNA syntheses were performed by reverse transcription of 1 µg of total RNA using SuperScript II Reverse Transcriptase (Invitrogen, Carlsbad, CA). PCRs were carried out for mouse A1R, A2AR, A2BR and A3R as well as for the housekeeping gene glyceraldehyde -3-phosphate dehydrogenase (GAPDH), Gα14 and GαGust. The presence of Gαgust in CV samples but not in samples of non-taste epithelium confirmed the specificity of the tissue dissection. Primer sequences for each PCR are listed in Table 1.

**Table 1:** Primers and Gene Information for PCR. Gα14 and Gαgust primer sequences were taken from [26].

*Gene*	Forward primer sequences	Reverse primer sequences	GeneBank Accession No.	*PCR Product Size*
*A1R*	CTTCTACCTGATCCGCAAGC	AAGGCTGAGGAGGAACAGTG	NM_001039510	470 bp
*A2AR*	CACGCAGAGTTCCATCTTCA	GAGAGGATGATGGCCAGGTA	NM_009630	564 bp
*A2BR*	GCTATGATCGTGGGCATTTT	TTTCCGGAATCAATTCAAGC	NM_007413	544 bp
*A3R*	GAAGCCCTGACTCTGTTTGC	CATCTTGACTTGCAGGCTGA	NM_009631	551 bp
*GAPDH*	CGTAGACAAAATGGTGAAGGTCGG	GCCAAAGTTGTCATGGATGACC	NM_008084	511 bp
*Gα14*	TCATGCAACAGAGGGACTTG	AGGGCCATGCTCAATTACAC	NM_008137	256 bp
*G*α*gus*	GCAACCACCTCCATTGTTCT	AGAAGAGCCCACAGTCTTTGAG	NM_001081143	286 bp
**Nested PCR Primers**				
*A1Rnest*	CAGAAACCCAGCATCCTCAT	AAGTTCCGGGTCACCTTTCT	NM_001039510	110 bp
*A2ARnest*	GAAGCAGATGGAGAGCCAAC	CACCTTCTTCTGCTCCACGT	NM_009630	161 bp
*A2BRnest*	CCTGTCACATGCCAATTCAG	TCTGGCCTTTTGGAGAAGAA	NM_007413	196 bp
*A3Rnest*	GGACTGGCTGAACATCACCT	TTGTCTCCCTAGCACTGGCT	NM_009631	148 bp

PCR amplifications for A1R, A2AR, A2BR, A3R, and GAPDH were performed under the following conditions: 94°C for 30 sec, 56°C for 30 sec, 72°C for 30 sec for a total of 35 cycles and elongation step at 72°C for 10 min. Gα14 and Gαgust had different annealing temperatures, 60°C and 58°C respectively. A total of 100 ng/µl of each cDNA sample was used in the PCR amplification. The reverse transcriptase step was omitted in controls to confirm removal of all genomic DNA. Amplification products were analyzed on 2% agarose gels and visualized with GelRed (Biotium, Hayward, CA). All PCR products were gel-purified (S.N.A.P. Gel Purification Kit, Invitrogen), cloned into pGEM-T easy cloning vector (Promega) and transformed into Mach1-T1 chemically competent cells (Invitrogen). Plasmid DNAs were purified using concert plasmid preparation kits (Invitrogen) and sequenced with Thermo Sequenase II dye terminator cycle sequencing kits (Amersham-Pharmacia). The sequence reactions were analyzed by an ABI373S DNA sequencer (Perkin Elmer). Nested PCR was also performed to ensure PCR results were correct products. Primer sequences for nested PCR are given in Table 1. These amplifications were performed under the following conditions: 94°C for 30 sec, 58°C for 30 sec, 72°C for 30 sec for a total of 30 cycles and elongation step at 72°C for 10 min. Controls in which Reverse Transcriptase were omitted showed no PCR product indicating lack of contamination by genomic material.

### *In situ* hybridization

Adult C57BL/6J mice were deeply anesthetized with chloral hydrate or sodium pentobarbital (injected i.p.) and perfused transcardially with 4% paraformaldehyde (PFA) in 0.1 M phosphate buffered saline (PBS). After postfixation (1–3 h) and cryoprotection in 20% sucrose in 0.1 M PBS overnight, tissues were sectioned longitudinally or transversely (12 µm) onto Superfrost Plus slides (Fisher Scientific, Hampton, NH). The frozen sections of CV and foliate (FO) papillae obtained from five C57BL/6J mice were hybridized with digoxigenin (DIG)-labelled antisense riboprobes corresponding to a partial cDNA of A2BR which were obtained by RT-PCR experiments using primers to the 5′ UTR region of each mRNA of interest. Sections were treated in 4% PFA in PBS for 10 min and washed in PBS. For partial proteolysis, sections were permeabilized with 1 µg/ml proteinase K in PBS at 37°C for 10 min, followed by a 5 min wash in PBS. Sections were refixed in 4% PFA for 10 min. For neutralization of free formaldehyde residues, sections were washed in 2 mg/ml glycine in PBS for 5 min. Endogenous alkaline phosphatase activity was quenched with 0.2 M HCl for 30 min, followed by two 5 min washes in PBS. Hybridizations were carried out at high stringency (50% formamide, 10 mM Tris-HCl, pH 7.6, 200 µg/mL tRNA, 1× Denhardt's solution, 600 mM NaCl, 0.25% SDS, and 1 mM EDTA, pH 8.0) at 58°C overnight. After hybridization the sections were washed twice in 5×SSC at 50°C for 30 min. Sections were then subjected to two high-stringency washes: one in 2×SSC at 50°C for 1 hr, followed by a 20 min wash in 0.2×SSC at the same temperature. To reduce background signals the sections were treated with RNase A solution (0.2 mg/mL; Sigma, St. Louis, MO) at 37°C for 30 min and then washed in 2× SSC at 50°C for 60 min and in 0.2× SSC for 60 min. For detection, signals were developed using alkaline phosphatase conjugated Fab fragments to DIG and standard chromogenic substrates (Roche Applied Science). To detect DIG-labeled hybridized RNA, an alkaline phosphatase (AP)-conjugated anti-DIG Fab fragment antibody (1∶500; Roche) was used. After washing in TNT buffer (100 mM Tris-HCl, pH 7.5, 150 mM NaCl, and 0.1% Tween 20) the sections were incubated with 4-nitro blue tetrazoliumchloride and 5-bromo-4-chloro-3-indolylphosphate (both from Roche) as chromogenic substrates.

### X-Gal Histochemistry

A2BR-lacZ mice were deeply anesthetized with sodium pentobarbital (injected i.p.) and perfused transcardially with 4% PFA in 0.1 M phosphate buffer. Extracted tissue was post fixed no more than 30 min, followed by two 15 minute washes in 0.1 M phosphate buffer (pH 7.4) containing 2 mM MgCl_2_, 5 mM EGTA, 0.01% sodium deoxycholate and 0.02% Nonidet-40 (buffer B). Tissue was then placed in an X-Gal solution (buffer B with 5 mM potassium ferricyanide, 5 mM potassium ferrocyanide, and 1 mg/ml X-gal). Optimal staining in the tongue was obtained after 24 hours at 37°C, at which time the tissue was washed in buffer B for 30 min and placed in PBS. For long-term storage, the tissue was moved to 4% PFA and placed at 4°C.

### Immunohistochemistry

#### Single immunohistochemistry

C57BL/6J mice were deeply anesthetized and perfused with 4% PFA in 0.1 M phosphate buffer. Following cryoprotection, frozen sections of CV papillae were obtained and prepared for immunohistochemistry. The A2BR antibody (1∶100 dilution, rabbit anti-A2BR; AB1589P, Chemicon International) used was generated against the synthetic peptide ^150^ATNNCTEPWDGTTNES^165^. This commercial antibody is directed against the amino acid sequence corresponding to the 2^nd^ extracellular domain (16 amino acids) of the human A2BR receptor gene and has only moderate sequence similarity (62.5% identity, 81.25% similarity) to the mouse A2BR sequence (ATSNCTELGDGIANKS). We established the specificity of this antiserum by comparing reactivity of WT to A2BR KO lines.

#### Double label immunohistochemistry

Because the antibody label was weak, albeit specific, we also utilized A2BR-driven expression of β-gal as an index of A2BR expression in A2BR-lacZ mice. For double localization studies, we detected the β-gal by immunocytochemistry using a guinea pig anti-β-gal antibody (1∶500) previously generated against full length β-gal by our laboratory ([44]).

Detection of other substances relied on primary antisera generated either in rabbit (rb) or goat (gt): rb anti-PLCβ2 (1∶1000, SantaCruz SC-206; directed against amino acid (aa) 1170–1181 of human origin), rb anti-Gαgust (1∶1000, Santa Cruz SC-395; directed against aa 93–112 of Gαgust of rat origin), rb anti-NCAM (1∶500, Chemicon Ab 5032; directed against purified chicken NCAM), rb anti-5HT (1∶5000; Immunostar # 20080), gt anti-Car4 (R&D Systems, # AF2414 directed against recombinant mouse Car4 ectodomain [residues 18 277]),or rb Gαq/11 (1∶1000; Santa Cruz sc-392; directed against peptide VFAAVKDTILQLNLKEYNLV near the C-terminus in mouse, but which cross-reacts with Gα14, [28].

All double-label assays were carried out coincidentally, since the primary antisera are derived from guinea pig (anti-β-gal) and another species, either rabbit or goat. Sections were placed overnight in primary antiserum cocktail diluted in blocking solution. Following three washes in buffer, the sections then were placed in a cocktail of secondary antisera (all diluted 1∶400) containing an anti-gp to detect the anti-B-gal (Alexa488-conjugated goat anti-guinea pig IgG [Molecular Probes] or donkey anti-guinea pig Dylight 549 [Jackson ImmunoResearch]) along with another secondary antiserum, either anti-rb or anti-gt according to the primary antisera employed: (Alexa546-conjugated goat anti-rabbit IgG [Molecular Probes], or donkey anti rabbit Alexa Fluor 488 [Invitrogen]). For the Car4 primary antiserum (made in goat) we utilized donkey anti-guinea pig Dylight 549 (Jackson ImmunoResearch) or donkey anti-goat Alexa Fluor 488 (Invitrogen) as secondary antisera. Some sections were counterstained with a fluorescent Nissl stain (NeuroTrace 640/660; Invitrogen).

The specificity of the secondary antisera was confirmed by omitting one or both primary antisera from the primary antiserum cocktail and finding no cross reactivity with the two applied secondary antisera. The specificity of β-gal immunoreactivity (IR) was determined by substitution of buffer for the primary antibody; this antiserum also yields no staining of WT mice, i.e. those lacking β-galactosidase. Previous papers from our laboratory showed the staining by the anti-Gαq/11 antibody is due to neither Gαq nor Gα11 leaving only Gα14 as the possible source of the immunoreactivity in taste buds [28]. We have subsequently confirmed that this antibody shows almost no staining of CV taste tissues from Gα14 KO animals (data not shown). In the text below, we refer to this antiserum as Gαq/11/14.

Photomicrographs were acquired using a monochrome Q-imaging camera on an Olympus BX41TF microscope and Q Capture software (QImaging). Brightness and contrast were adjusted in Photoshop®. Laser scanning confocal images were captured on a Olympus Fluoview confocal laser scanning microscope (LSCM) FV300 (Olympus Corporation) using sequential scanning of the different channels to avoid inappropriate side-band excitation of fluorophores. For some confocal images, the median filter was applied to eliminate high frequency pixel noise associated with confocal acquisition. Images were assembled in Photoshop and brightness and contrast levels were adjusted to obtain an image presentation closely mirroring the visual appearance of the tissue.

#### Cell Counting

In order to estimate the degree of coincident expression in double label immunostained sections, we counted the number of taste cells stained by each marker in sections through the circumvallate papillae of two A2BR-lacZ mice. The appearance of label in these mice was not obviously different than in the many other mice – both WT and A2BR-lacZ – examined during the course of this study. A taste cell was considered labeled if it exhibited label above background levels and for which a clear nuclear profile was evident, i.e. cell profiles not containing the nucleus were not counted. For each marker, either 7 or 8 sections were examined and all taste buds (n>100 for all markers) in those samples were counted. For those markers for which no co-localization was evident (e.g. Car4, 5HT, NCAM) counts were not made but a minimum of 100 taste buds were examined.

### Chorda tympani and glossopharyngeal nerve recordings

Recordings from intact taste nerves were performed as described previously by Finger et al. [1]. Briefly, WT and A2BR-KO mice (8–10 wks old, 20–23 g) were anesthetized and maintained under surgical level of anesthesia with sodium pentobarbital (50 mg/kg). Toe-pinch was used to determine the depth of surgical anesthesia. The trachea was cannulated and the animal was allowed to breathe room air via the tracheotomy during the procedure. The animal's head was held by a clamp; both the chorda tympani branch of the facial nerve (CN VII) and glossopharyngeal nerve (IX) were exposed using a ventral approach. The chorda tympani nerve (n = 6 KO, 6 WT) was dissected free and cut near the tympanic bulla. The glossopharyngeal nerve (n = 7 KO, 7 WT) was exposed by removing the underlying tissue and cut near its entrance to the posterior lacerated foramen. Then, the dissected free nerve was placed over a platinum-iridium wire hook for recording. A ground electrode was placed in a nearby muscle. The nerve signal was acquired at 200 Hz and integrated with a PAVC-1 integrator (Duck Engineering Design) with a time constant of 1 s. Integrated nerve recording data were collected using a MP100 hardware interface to Acknowledge 3.8 software (BIOPAC, Santa Barbara CA). Fungiform (FU), CV and FO papillae were exposed by a small suture sewn in the tip of the tongue. Taste stimuli were dissolved in distilled water and delivered to the tongue under constant flow by a Valvelink 16 perfusion system (Automate Scientific, Inc. San Francisco, CA). Most mice were tested for tastant-induced nerve responses to 100 mM, 200 mM, 300 mM, 500 mM, and 1000 mM sucrose, 500 µM SC45647, 30 mM sucralose, 100 mM NaCl and 30 mM quinine. Taste solutions were applied to the tongue for 1 min followed by a rinse with distilled water. All solutions including distilled water were maintained at temperatures between 33 to 37°C. Data analysis was performed by obtaining the area under the curve for 60 s of the integrated nerve response to each taste stimulus. The data were normalized to 100 mM NH_4_Cl responses to reduce variability across recordings. For purposes of illustration, integrated responses in glossopharyngeal recordings were smoothed, using a mean value of 30 points in the Acknowledge software. The normalized nerve responses were compared between WT and A2BR-KO mice using one-way ANOVA with Bonferroni's Multiple Comparison Test (p<0.05; GraphPad Prism 5, La Jolla CA).

## Results

### Expression of adenosine receptors in taste epithelium

**Figure 1:**
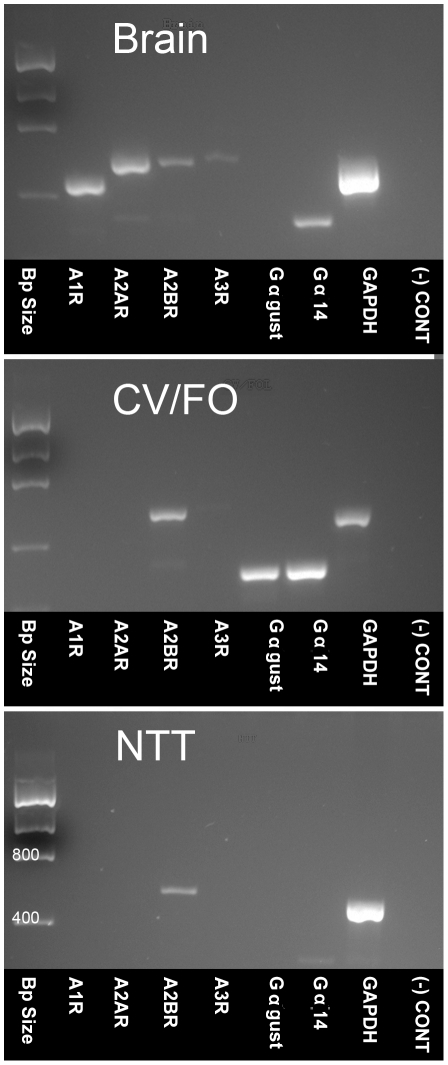
RT-PCR reveals expression of adenosine receptors in brain, taste epithelium but limited expression in non-taste lingual epithelium. **Top:** In the brain, all 4 adenosine receptors (A1R, A2AR, A2BR and A3R) are readily detected along with Gα14. Gαgust is not detectable. **Center:** In posterior taste fields (CV/FO) A2BR transcript is detected; a faint band is found for A1R in some preparations. Both Gαgust and Gα14 are also evident. **Bottom:** In non-gustatory epithelium (NTT = Non-Taste Tissue) A2BR but not Gαgust or Gα14 is detected. A faint band for A1R is also present in some preparations as in CV/FO epithelium. PCR products were visualized using UV illumination following ethidium bromide staining. Expression of mRNA for GAPDH was used as a positive control. The reverse transcriptase step was omitted as a negative control [(-)CONT] to confirm removal of all genomic DNA.

To test for the presence of mRNAs for adenosine receptors, we used RT-PCR in mouse CV papillae along with whole brain and non-gustatory tongue epithelium. The amplification products were of the expected size and sequence in all tissues where they were detected. RT-PCR analyses showed that mRNAs for all adenosine receptors were readily apparent in brain mRNA. Samples from CV papillae displayed a robust signal for A2BR and occasionally a faint band for A1R, but no detectable signal for either A2AR or A3R (Fig. 1). Nested PCR confirms the identity of all specific bands reported. A similar pattern was seen for the non-taste epithelium. No specific products were detected when the reverse transcriptase was omitted from the reverse transcription mixture, indicating the absence of contamination by genomic DNA.

**Figure 2:**
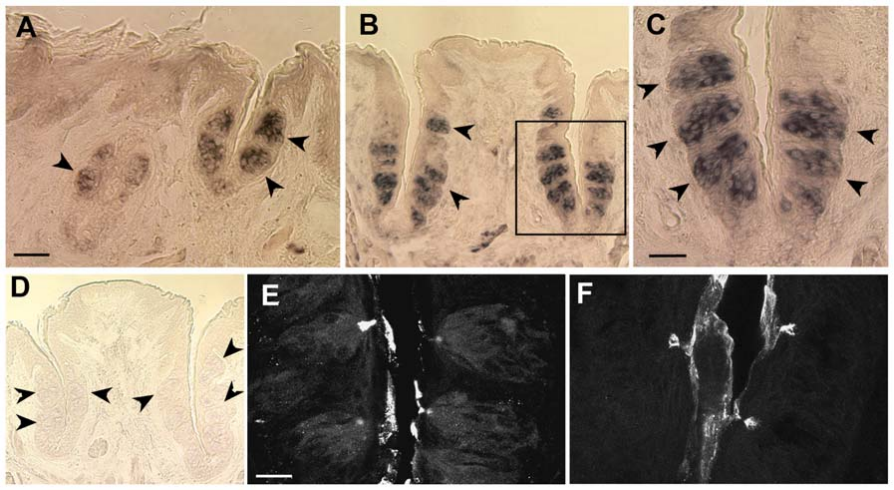
In situ hybridization and immunocytochemistry show restricted expression of A2BR in posterior taste epithelia. The FO (**A**), CV (**B** and **C**) papillae show expression of A2BR mRNA in taste buds (arrowheads). Panel C is high magnification of boxed area in **B**. No signal is detectable in CV taste buds (arrowheads) when the section was hybridized with the sense probe (**D**). Our data show that immunoreactivity for A2BR was seen only weakly in CV taste buds of WT mouse (**E**). The CV taste buds of A2BR KO/LacZ mouse did not react with A2BR antibody (**F**), except for non-specific trapping of the antibodies at the surface of the epithelium and in taste pores. Scale bars = 50 µm in **A** (also applies to **B** & **D**); 20 µm in **C** & **E** (also applies to **F**).

Since RT-PCR showed the presence of A2BR mRNA in both taste and non-taste epithelia, we used *in situ* hybridization to investigate the expression patterns of the A2BR genes in sections of mouse FO and CV papillae. As shown in Fig. 2 (A, B and C), A2BR expressing cells were clearly detectable in FO, CV taste bud cells, whereas they were not observed in the other taste epithelia. Further, no A2BR mRNA signal was observed in non-taste epithelium.

We then sought to determine expression at protein level. The rabbit anti-A2BR antibody (AB1589P; Chemicon International) gave only weak, but repeatable staining for A2BR in CV taste buds of the C57BL/6J mouse (Panel E in Figure 2). As a negative control, we tested the same antibody in A2BR KO mouse. Mice homozygous for this transgene have no intact coding sequence for A2BR protein [42] and A2BR immunostaining was not seen in taste buds (Panel F in Figure 2) suggesting specificity of the antiserum. Residual signal in taste pores and trench wall epithelium in the A2BR KO tissue is the same as in WT, suggesting some non-specific background binding of the A2BR antibody to these tissue elements.

**Figure 3:**
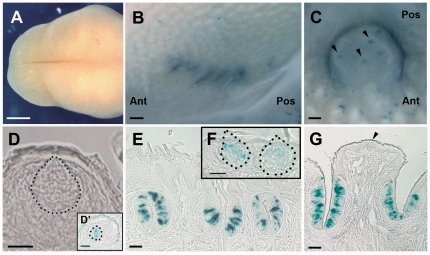
β-galactosidase (X-gal)-reacted A2BR-KO/LacZ mouse tissues show staining of posterior but not anterior taste buds. The dorsal surface of tongue where fungiform papillae lie, does not show any blue spots (**A**). Blue staining along the lateral margin and posterior midline of the tongue shows A2BR KO/LacZ respectively in FO (**B**) and CV (**C**) papillae. Black arrowheads indicate taste buds of the top of CV (**C** and **G**). Longitudinal sections through an X-gal-treated FU (**D, D′**), FO (**E**), palate (**F**), and CV (**G**). Dotted lines indicate the outline of FU (**D** and **D′**) and palatal (**F**) taste buds. In nearly all cases X-gal does not react with FU taste buds (**D**). An exception is shown in **D′**. **Ant** and **Pos** indicate the anterior and posterior directions of tongue (**B** and **C**). Scale bars = 1 mm in A; 100 µm in B, C, E, G; 20 µm in D, D′, F.

In order to confirm the pattern of label produced by the A2BR antiserum, we examined lingual tissue from A2BR-KO/(homozygous) and A2BR-lacZ (hemizygous) mice with X-gal staining or anti-β-gal antibody. Both the A2BR-KO and A2BR-lacZ mice show the endogenous A2BR gene promoter-dependent expression as β-gal protein in various tissues. As revealed by X-gal staining, strong positive reaction is visible in CV and FO papillae taste buds (Fig. 3B, C, E and G). Blue staining was rare in FU and palate taste buds (Fig. 3 A, D, D′, F); almost all taste buds in these fields were devoid of β-gal reaction product. In order to assess in detail β-gal (A2BR) expressing taste cells in the posterior taste fields, we undertook immunohistochemical analysis using antibodies against β-gal. The immunopositive cells constitute less than half of the population in any given taste bud. In non-taste epithelium immunostaining for β-gal (A2BR) is negative except for occasional small blood vessels. The presence of A2BR in these vessels likely accounts for the positive signal obtained with PCR of non-taste epithelium.

### Differential expression of A2BR in taste cells

**Figure 4:**
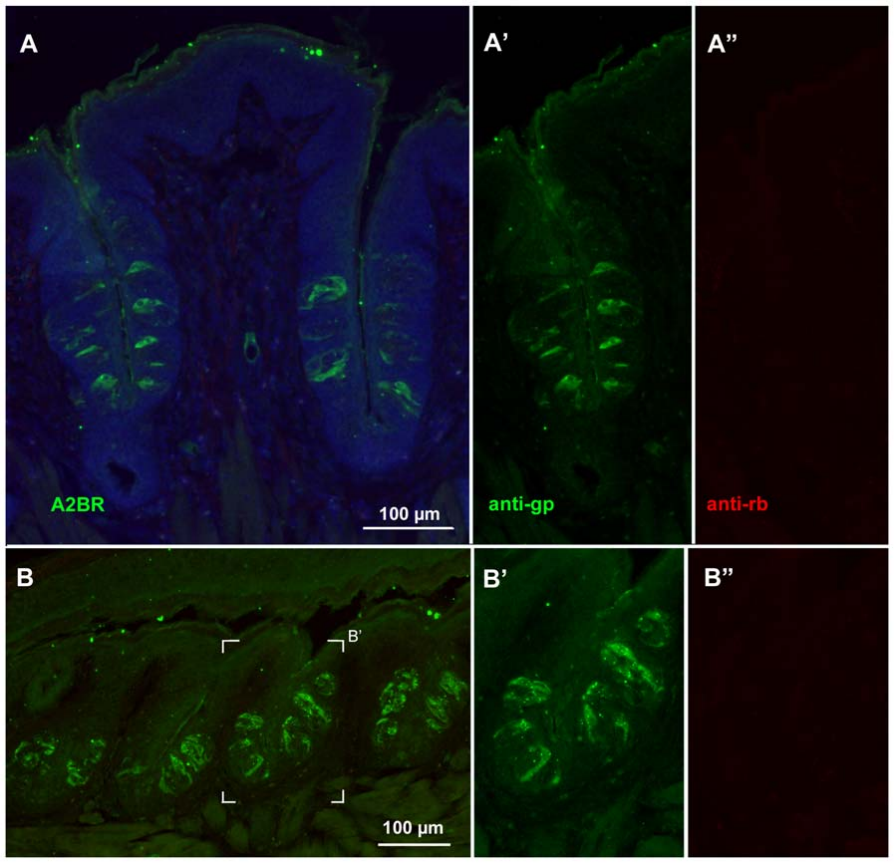
Specificity of the secondary antisera is demonstrated by selective immunostaining of taste epithelia. Immunostaining of CV (**A**) and foliate (**B**) papillae from an A2BR-βgal mouse using only the gp-anti-βGal primary antiserum but applying both anti-gp and anti-rb secondary antibodies. Although prominent immunoreactivity is evident with the anti-gp secondary antiserum (green: **A, A′** and **B, B′**), no cross-reactivity is evident from the anti rb secondary (red: **A, A″** and **B, B″**). Thus the rb-secondary antiserum is appropriate for use in co-localization studies illustrated in Figs. 5–8. In the top row, panels **A′** and **A″** show only the left half of panel A. In panel B, the white frame marks show the area enlarged in panels **B′** and **B″**. These low power micrographs also show the relative abundance of A2BR-βgal taste cells in the two taste fields.

**Figure 5:**
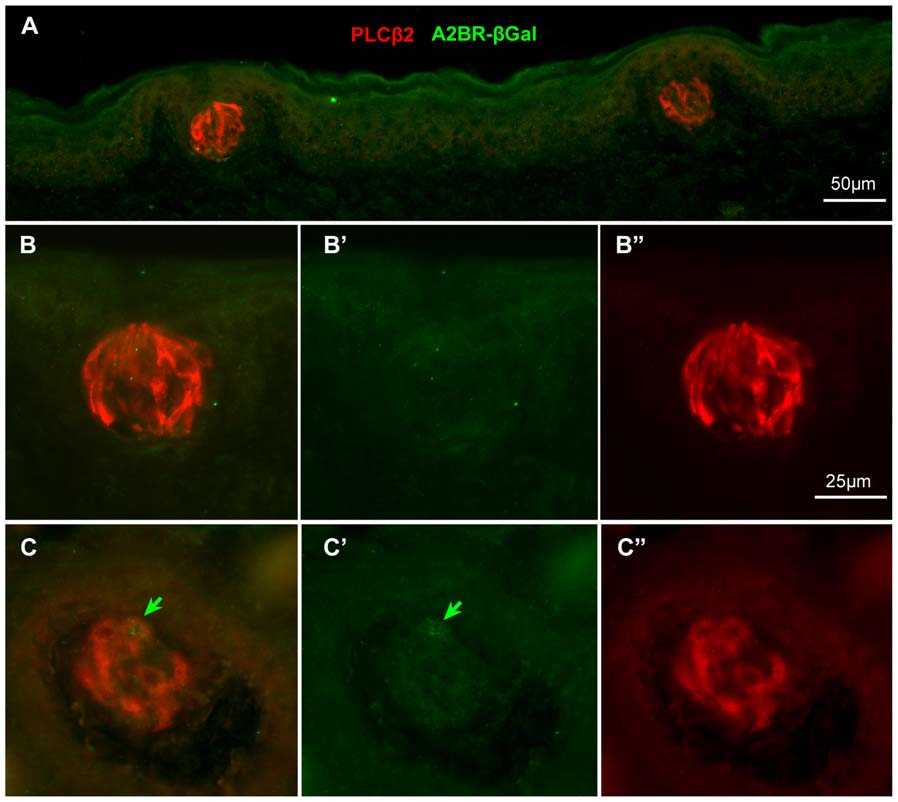
Double immunolabeling for PLCβ2 (red) and βgal (green) in the palate and fungiform taste fields where A2BR-βgal expression is weak or absent shows specificity of the anti-gp secondary antiserum as well as lack of staining for βgal. Panels **A** and **B** show palatal taste buds; the lack of green staining (**A; B, B′**) shows both the absence of βgal immunoreactivity and the lack of cross reactivity of the anti-gp secondary antiserum with the rb PLCβ2 primary antiserum plainly visualized with the red anti-rb secondary antiserum (**B, B″**). Panel **C** shows a fungiform taste bud where only one (green arrow) of the PLCβ2-positive cells (red, **C, C″**)) shows faint reaction for βgal (green, **C, C′**). The lack of green label in the other strongly positive red cells again demonstrates specificity of the anti-gp (green) antiserum. B′, B″ and C′, C″ show color separation images of panels B and C respectively. Scale bar in B″ also applies to C.

In order to test whether A2BR-driven β-galactosidase was associated with particular subpopulations of taste cells, we undertook a series of double-label immunocytochemical experiments using antisera directed against marker molecules for the various cell types. For these experiments to be reliable, it is necessary to demonstrate lack of cross-reactivity of secondary antisera with antisera raised in the other species, i.e. to show that the anti-rabbit secondary did not cross-react with guinea pig primary antisera and vice versa. In no case did we observe cross-labeling of a primary antiserum with the inappropriate secondary antiserum (Fig. 4). The specificity of the secondary antisera also was evident in tissues from fungiform and palatal taste fields where taste buds rarely exhibit β-gal immunoreactivity, i.e. the absence of label by one secondary in the presence of label by the other secondary shows specificity of the double labeling (Fig. 5).

**Figure 6 pone-0030032-g006:**
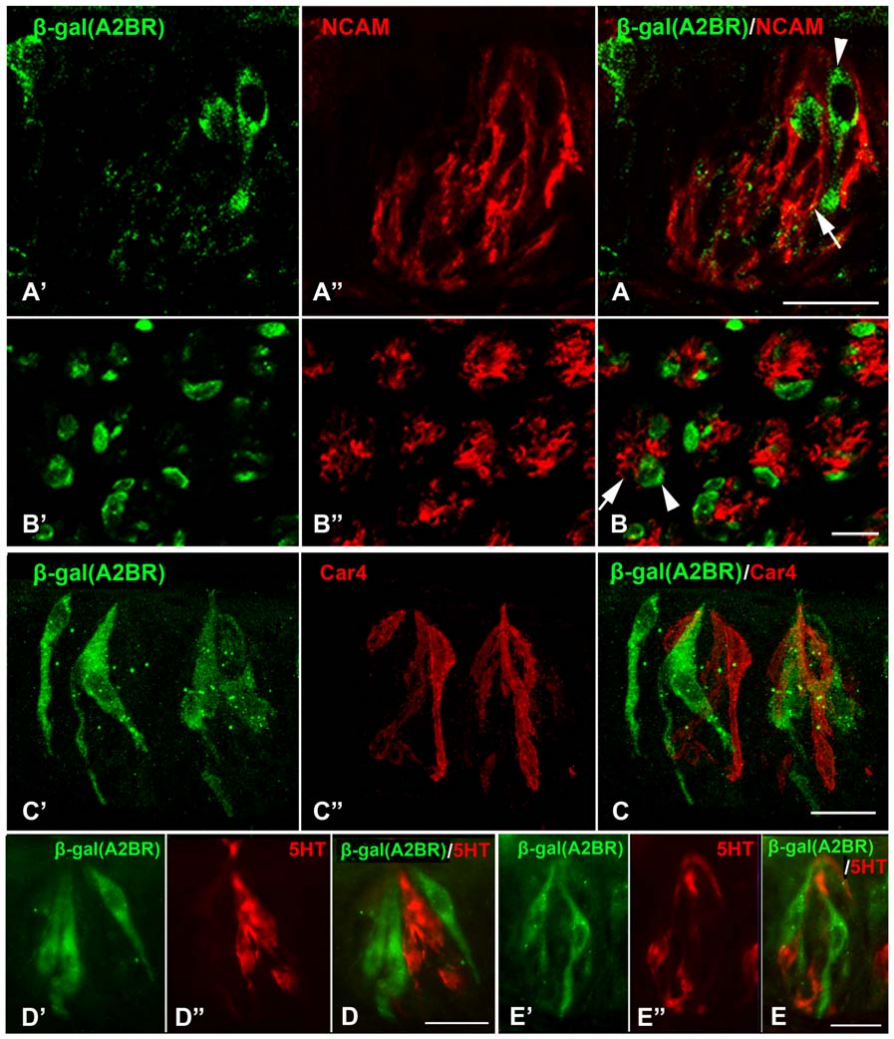
No A2BR-positive taste cells are Type III taste cells. Confocal laser scanning microscopy images of double-labeled sections of CV taste buds stained for markers of Type III cells (NCAM, Car4, 5HT = red). Taste cells positive for β-gal (A2BR, **A**, **B** arrowheads) did not show NCAM-IR (**A**, **B** arrows). Similarly, taste cells positive for Car4 (red in **C″ and C**) never co-label for A2BR (green in **C′ and C**). Likewise, conventional epifluorescence images of taste cells in CV (**D**) and foliate (**E**) papillae show separate label for A2BR (green in **D′, D, E′** and **E**) and 5HT (red in **D″, D, E″** and **E**). Scale bars = 20 µm.

Labeling for A2BR (β-gal) correlates with markers of taste cell type and function. Double immunostaining with β-gal (A2BR) and markers of Type III taste cells, NCAM (Fig. 6 A, B), Car4 (Fig. 6C) and 5HT (Fig. 6 D, E), showed no obvious overlap between β-gal (A2BR)-IR and any marker of Type III cells (Fig. 6). Similarly, in the hundreds of taste buds examined, no taste cells showing immunoreactivity for type III cell markers was immunoreactive for β-gal.

**Figure 7 pone-0030032-g007:**
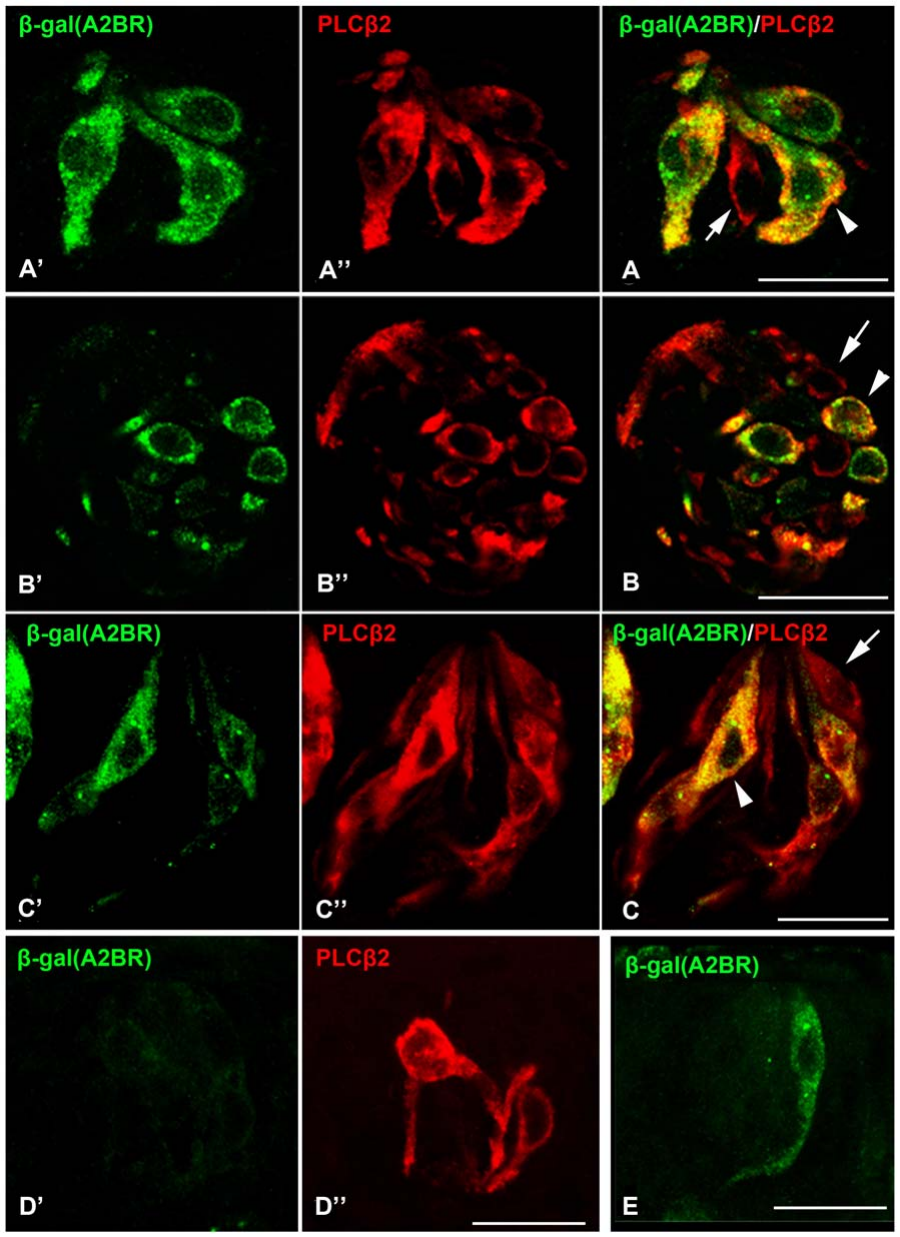
All A2BR-positive taste cells are Type II cells, indicated by expression of PLCβ2. Confocal laser scanning microscopy images of double-labeled longitudinal (**A′, A″, A**) and cross sections (**B′, B″, B**) of FO taste buds stained for PLCβ2. Although all A2BR-positive cells express the global Type II cell marker, PLCβ2, a subset of PLCβ2-IR cells does not show β-gal (A2BR)-IR (**B, C**, arrows). Similarly, in CV papillae, all A2BR taste cells (green **C′ and C**) express PLCβ2 (red in **C″ and C**) but not vice versa (arrow in panel **C**). Images of FU taste buds show little β-gal (A2B-R)-IR (green in **D′**) although PLCβ2 expression (red **in D″**) is abundant. Rarely, single cells in FU exhibit immunoreactivity for A2BR-driven β-gal as shown in panel **E**. Scale bars = 20 µm.

In contrast, many cells in posterior lingual taste buds that display the characteristic marker of Type II taste cells, PLCβ2-IR [25], also express A2BR-driven β-gal (Fig. 7 A, B, C arrowheads). Further A2BR-expressing taste cells have the classic morphological appearance of Type II taste cells, most notably a large, round nucleus. However, not all PLCβ2-IR taste bud cells were β-gal (A2BR)-positive (Fig. 7A, B, C arrows) indicating that A2BR is expressed in only a subset of Type II taste cells. A quantitative analysis of this co-expression pattern shows that all cells expressing A2B-driven β-gal co-express PLCβ2 (see Table 2) but only 43.8% of PLCβ2-IR cells express A2B-driven β-gal (n = 288). Little or no β-gal (A2BR) IR was detected in FU or palatal taste buds (Fig. 7 D′, D″; E).

**Table 2:** Co-expression of A2BR-lacZ and Type II Taste Cell Type Markers.

A2BR only	Double Label	PLCß2-only	% A2BR co-localization with marker
0	126	162	43.8%
A2BR only	Double Label	Gustducin-only	% A2BR co-localization with marker
94	14	104	13.0%
A2BR only	Double Label	Gq/11/14 only	% A2BR co-localization with marker
2	132	55	98.5%

**Figure 8:**
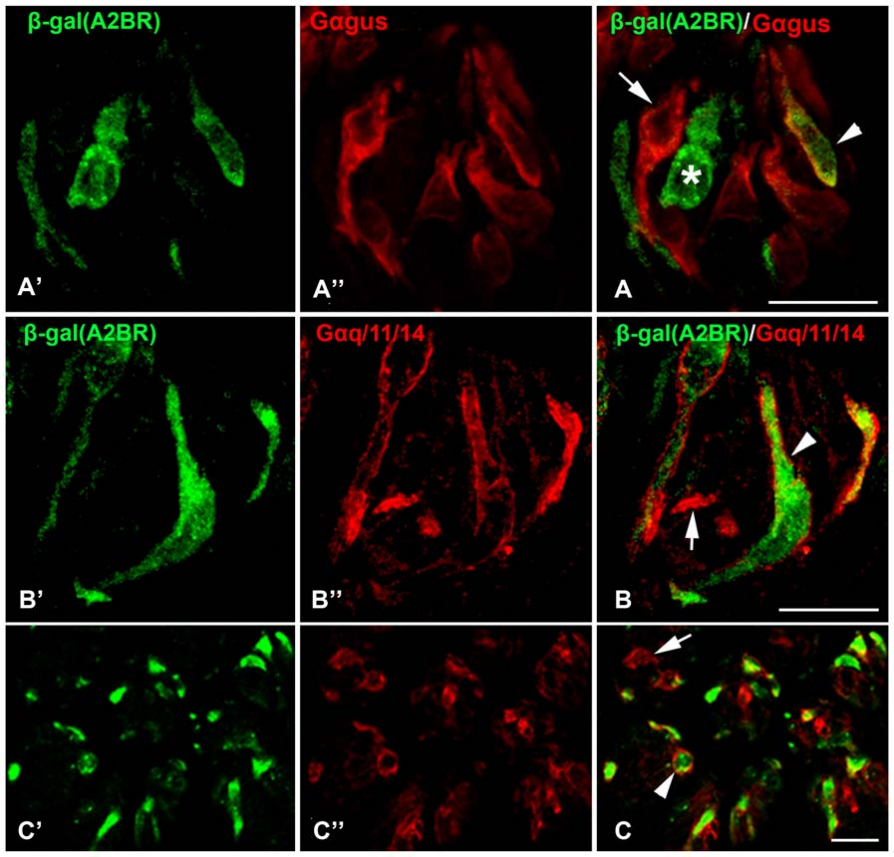
A2BR expression associates with expression of Gα14 but not Gαgust. Confocal laser scanning microscopy images of double-labeled longitudinal and cross sections of CV taste buds stained for markers of subsets of Type II cells. Gαgust, (**A″, A**) co-labels only a small percentage of cells expressing β-gal (A2BR, green in **A′**, arrowhead in **A**), but not all cells positive for Gαgust showed β-gal (A2BR)-IR (arrow in **A**). Also some β-gal (A2BR)-IR cells did not show Gαgust-IR (**A**, asterisk). In contrast, almost all (98.5%; see Table 2) β-gal (A2BR)-IR cells co-localized with Gq/11/14-IR (**B and C**, arrowheads). Not all Gαq/11/14-IR cells show β-gal (A2BR)-IR (**B and C**, arrows). The arrow in panel **B** indicates the basal process of a single label taste cell. This profile, nearly 2 µm across is too large to be an intragemmal nerve process. Scale bars = 20 µm.

Type II cells can be subdivided according to their expression of taste receptors and their associated G proteins. Gαgust-IR taste cells are a subset of Type II cells [27], [45]. In posterior tongue, Gαgust predominantly marks bitter-responsive taste cells with lesser expression in the umami and sweet sensitive populations [28], [29]. Double immunolabeling with β-gal (A2BR) and Gαgust revealed incomplete overlap of immunoreactivity in both CV and FO papillae (Fig. 8A). Few (14 of 118) taste cells that expressed Gαgust-IR were positive for β-gal (A2BR)-IR (Table 2 and Fig. 8A, arrowhead). Similarly, nearly all (94 of 108) β-gal (A2BR)-IR taste bud cells lacked Gαgust-IR (Table 2 and Fig. 8A, asterisk). Two previous studies demonstrated that in posterior lingual taste fields, all taste cells that express the sweet receptor T1R2/T1R3, also express Gα14 ([28], [29]). Accordingly, we used Gα14 as a marker for sweet-receptor expressing taste cells in circumvallate and foliate papillae. Comparison of A2BR-IR and Gα14-IR staining in taste buds (Gαq/11/14-IR) showed over 98% (132 of 134) A2BR-IR taste bud cells co-express Gα14-IR (Table 2; Fig. 8 B, C) although only 70.6% of the Gq/11/14 positive population expressed A2BR-driven β-gal.

In summary, numerous β-gal (A2BR)-IR taste bud cells were detected in the posterior lingual taste fields, but few were present in taste buds of FU papillae. The β-gal (A2BR)-IR taste cells showed markers of Type II (PLCβ2), but not Type III (NCAM, Car4 and 5HT) taste cells. Within the Type II cell population the A2BR-IR expressing cells usually did not co-express Gαgust, the gustatory G protein associated with bitter-responsive taste cells of the posterior tongue, but did express Gα14, which is co-expressed there with the sweet taste receptor T1R2/T1R3 [28], [29]. Taken together, these anatomical results suggest close correspondence between expression of A2BR and the sweet taste transduction cascade. Accordingly we undertook physiological experiments to test whether genetic deletion of A2BR specifically affects responses to sweet in the posterior taste fields.

### A2BR-dependent sweet taste responses as revealed by gustatory nerve recording

**Figure 9:**
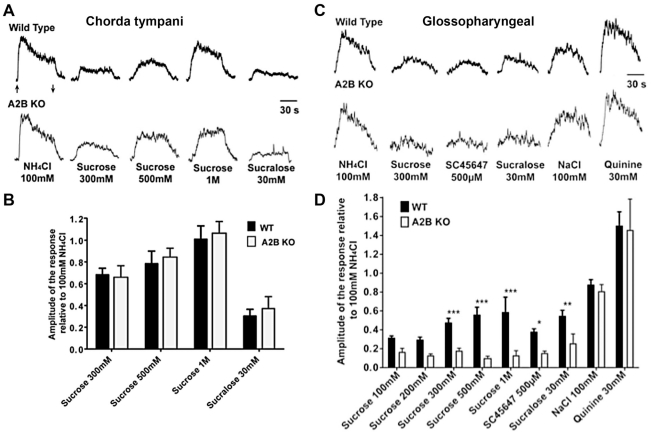
A & B. Gustatory nerve recordings from A2BR-KO mice show specific loss of sweet responses from the posterior but not anterior taste fields. Chorda tympani nerve recordings from A2BR KO and WT mice. (**A**). Integrated nerve responses from WT (top) and A2BR KO (bottom) mice to 300 mM, 500 mM, 1 M sucrose and 30 mM sucralose. (**B**) Bar graph (mean±SEM) represents comparison of the amplitude of the response between WT (black) and A2BR KO (white) mice (n = 4–6 mice for each stimulus). ANOVA revealed no statistical differences between genotypes for these records from the chorda tympani nerve. **C & D.** Glossopharyngeal nerve recordings from WT (top) and A2BR KO (bottom) mice. (**C**). Integrated nerve responses from WT and A2BR KO mice to taste stimulation. (**D**). Relative amplitude of the response between WT (black bars) and A2BR KO (white bars) to 100 mM, 200 mM, 300 mM, 500 mM, 1 M sucrose, 500 µM SC45647, 30 mM sucralose, 100 mM NaCl and 30 mM quinine **B & D.** Each integrated taste response was normalized to the response to 100 mM NH_4_Cl. Asterisks indicate statistical significance between WT and A2BR KO (p<0.05; one-way ANOVA with Bonferroni's Multiple Comparison Test). Taste stimulation was applied for 60 s in all experiments; arrows in (**A**) denote onset of stimulus application and removal, which is the same for all traces. Each animal for each group received the same taste stimulation and water rinses (n = 4–7 mice for each stimulus). Bars represent Mean±SEM.

To determine the functional role of the A2BR in taste buds, we performed whole nerve recording of both glossopharyngeal and chorda tympani nerves of A2BR KO mice. Since A2BR is expressed heavily in posterior but not anterior tongue, KO effects should be present in the glossopharyngeal (innervating CV and most FO papillae) but not chorda tympani nerve (innervating FU and few FO papillae) responses. Compared to WT mice, the magnitude of the response to all sweet compounds in KO mice was reduced in the glossopharyngeal (Fig. 9C, D) but not chorda tympani (Fig. 9A, B) nerve. In the glossopharyngeal nerve, responses to 300 mM, 500 mM and 1 M sucrose were significantly different between A2BR KO and WT mice (p<0.05). Similarly, responses to the synthetic sweeteners SC45647 (500 µM) and sucralose (30 mM) were also decreased in A2BR KO compared to WT mice (p<0.05). No differences were found in glossopharyngeal nerve responses to 100 mM NaCl and 30 mM quinine between KO and WT animals. In contrast, in the chorda tympani nerve, responses to 300 mM, 500 mM, 1 M sucrose and 30 mM sucralose were not different between A2BR KO and WT mice (Fig. 9A, B). These results are consistent with our β-gal and immunocytochemical expression analysis, showing significant expression of A2BR in sweet receptor-expressing CV taste cells, but little or no expression of A2BR in FU taste buds.

## Discussion

Our experiments show that the adenosine receptor A2BR is selectively expressed in a subpopulation of taste cells in posterior lingual taste fields i.e. in those cells that express Gα14 which is indicative of cells that express the sweet taste receptor [28], [29]. Further, we demonstrate, using A2BR-KO mice, that glossopharyngeal nerve responses to all sweet compounds are severely depressed relative to WT mice. These data suggest that adenosine, a breakdown product of ATP signaling, is required for normal sweet taste function in posterior lingual taste fields. This is the first demonstration of a role for this low affinity adenosine receptor in sensory signaling. Previous studies in a variety of tissues have shown that activation of A2BR is most commonly associated with modulation of inflammatory responses, including in gut, blood vessels, and lung; for review, see [46]. In those systems, activation A2BR induces protective responses to the inflammatory or anoxic insults [42], [43], [47]–[50]. While some of these functions might be related to the role of A2BR in lingual epithelium, e.g. in control of mast cell secretion, the restricted distribution of expression in taste buds suggests a more discrete function related specifically to taste transduction.

In the taste system, activation of A2BR appears to enhance transmission of a sensory signal to the nervous system. In the brain and other sensory systems, adenosine modulates synaptic transmission by enhancing transmitter release, but these effects are mediated by either A1R or A2AR receptors [51], [52], never by A2BR.

In the murine taste system, A2BR is expressed only in posterior taste fields, i.e. CV and FO papillae. In contrast, in the macaque monkey, A2BR expression is detected at the mRNA level in both anterior and posterior tongue, i.e. CV and FU taste epithelia (Supplementary data, in [53]). Dando and co-workers [41] confirm that even at the mRNA level, A2BR expression in mice is limited to posterior taste fields. The difference in A2BR expression may reflect a difference in sweet transduction mechanisms between the species. In mice, expression of A2BR in posterior lingual taste buds coincided with expression of the G protein Gα14 that is co-expressed with the sweet receptor T1R2/T1R3 [28], [29]. In anterior lingual taste buds of mice, sweet receptors are largely co-expressed with Gαgust [28], [54], [55]. Whereas Gαgust is a member of the Gαi/o family, Gα14 is a member of Gαq family. Unclear, however, is whether the sweet receptor couples to Gα14 or merely exhibits co-incident expression. Several other Gαi/o family members are expressed in taste buds, and so the exact role of Gα14 in sweet taste signaling remains to be elucidated.

Our functional studies show that responses of the glossopharyngeal nerve to sweet stimuli, both sucrose and synthetic sweeteners, are significantly reduced in A2BR KO mice compared to WT mice. In contrast, chorda tympani responses to sucrose and synthetic sweeteners are similar in A2BR KO and WT mice. These data are consistent with our ß-gal staining, showing that A2BR expression is restricted to posterior taste fields innervated by the glossopharyngeal nerve. Further, our functional studies showed that the KO effect was restricted to sweet stimuli, which would be predicted from our double label immunohistochemical studies. Taken together, our functional studies suggest that the role of A2BR is to potentiate sweet taste responses. However, the intracellular mechanisms that mediate the A2BR response are not clear, since A2BR can couple to two different intracellular cascades; either Gαq, resulting in increases in intracellular Ca^2+^ or Gαs, resulting in increases in cAMP [56]. Since the A2BR is co-expressed with Gα14, it is possible that A2BR couples directly to Gα14, causing increases in intracellular Ca^2+^, which would tend to potentiate Ca^2+^ responses to sweet stimuli and thus potentiate ATP release. Alternatively, a Gαs mechanism cannot be ruled out, since Gαs is expressed in taste receptor cells [57], although whether it is co-expressed with sweet taste receptors has not been determined.

Regardless of the mechanism, our results suggest that adenosine, produced as a product of ATP signaling in response to sweet tastants, acts to potentiate sweet taste responses by activating A2BR. Recent data [41] provides direct support for this hypothesis. Using a lingual slice preparation, they showed that adenosine enhances Ca^2+^ responses to sweet stimuli. Further, utilizing isolated taste buds and biosensor cells expressing P2X receptors, they showed sweet taste evoked ATP release is increased by adenosine in the bath [41]. These data suggest that adenosine acts in an autocrine manner to potentiate sweet receptor signaling in posterior tongue.

Interestingly, caffeine, a non-selective antagonist of adenosine receptors, increases the perceived intensity of several synthetic sweeteners in humans [58], [59]. Since this effect is reversed by the application of adenosine, caffeine was hypothesized to act via adenosine receptors in taste buds. However, our data suggest that if caffeine is acting via adenosine receptors in humans as it does in mice, that it should decrease, rather than increase the perceived intensity of sweeteners, i.e. the effect of the antagonist (caffeine) should mimic the A2BR KO. Since caffeine increases the intensity of sweeteners in humans, this effect would seem to be mediated via a different mechanism.

In conclusion, we demonstrate a novel role for A2BR in transduction and transmission of sweet taste information. This adds a unique functionality to the large repertoire of protective responses previously described for this widespread, low-affinity receptor.
